# Real-Time Deep Learning-Based Drowsiness Detection: Leveraging Computer-Vision and Eye-Blink Analyses for Enhanced Road Safety

**DOI:** 10.3390/s23146459

**Published:** 2023-07-17

**Authors:** Furkat Safarov, Farkhod Akhmedov, Akmalbek Bobomirzaevich Abdusalomov, Rashid Nasimov, Young Im Cho

**Affiliations:** 1 Department of Computer Engineering, Gachon University, Sujeong-Gu, Seongnam-si 461701, Republic of Korea; safarov@gachon.ac.kr (F.S.); farhodsam92@gmail.com (F.A.); 2 Department of Artificial Intelligence, Tashkent State University of Economics, Tashkent 100066, Uzbekistan; rashid.nasimov@tsue.uz

**Keywords:** drowsiness detection, eye tracking, blinking, deep learning, computer vision, classification

## Abstract

Drowsy driving can significantly affect driving performance and overall road safety. Statistically, the main causes are decreased alertness and attention of the drivers. The combination of deep learning and computer-vision algorithm applications has been proven to be one of the most effective approaches for the detection of drowsiness. Robust and accurate drowsiness detection systems can be developed by leveraging deep learning to learn complex coordinate patterns using visual data. Deep learning algorithms have emerged as powerful techniques for drowsiness detection because of their ability to learn automatically from given inputs and feature extractions from raw data. Eye-blinking-based drowsiness detection was applied in this study, which utilized the analysis of eye-blink patterns. In this study, we used custom data for model training and experimental results were obtained for different candidates. The blinking of the eye and mouth region coordinates were obtained by applying landmarks. The rate of eye-blinking and changes in the shape of the mouth were analyzed using computer-vision techniques by measuring eye landmarks with real-time fluctuation representations. An experimental analysis was performed in real time and the results proved the existence of a correlation between yawning and closed eyes, classified as drowsy. The overall performance of the drowsiness detection model was 95.8% accuracy for drowsy-eye detection, 97% for open-eye detection, 0.84% for yawning detection, 0.98% for right-sided falling, and 100% for left-sided falling. Furthermore, the proposed method allowed a real-time eye rate analysis, where the threshold served as a separator of the eye into two classes, the “Open” and “Closed” states.

## 1. Introduction

Driver drowsiness detection is a significant concern for road safety. Drowsy driving is defined as driving when there are symptoms of fatigue, sleepiness, or an inability to maintain alertness. There are several factors underlying the feeling of drowsiness such as a lack of sleep, long driving hours, and monotonous conditions. The eye-blinking-based method is a promising approach to detect driver drowsiness. This process involves monitoring the pattern and frequency of eye-blinks while driving [1,2]. Eye-blinks are a good indicator of a driver’s level of alertness and are based on the frequency and pattern changes of eye-blinks with respect to the driver’s condition. A decrease in the frequency of eye-blinks or an increase in the duration of eye closure can be indicators of driver drowsiness. By monitoring these variable changes, it is possible to determine whether a driver is at risk of falling asleep at the wheel. This information can be captured using various systems and sensor technologies such as in-vehicle cameras or wearable devices to provide real-time monitoring of driver alertness and identify a person’s condition. The eye-blinking-based method is non-intrusive and can be used to detect driver drowsiness. The implementation of this method can significantly improve road safety by reducing the number of accidents [3,4,5].

Several studies have discussed methods to detect driver drowsiness based on eye-blink analyses, including the implementation of computer-vision techniques, machine-learning algorithms, and physiological signals [6,7,8,9,10]. Ref. [6] proposed the use of eye-blinks and yawning features to detect driver drowsiness. The authors investigated a system that captured video footage of a driver and analyzed the frequency and duration of eye-blinks and yawns to determine the level of drowsiness. They applied computer-vision techniques to extract relevant features from video sequences of drivers. The main contribution of this study was the integration of eye-blink and yawning features for the detection of drowsiness. One study found that a combination of these two features provided a more reliable indication of drowsiness than the solo implementation of eye-blink features. This was probably because yawning is a well-known physiological response to drowsiness and can provide complementary information for an eye-blink analysis. Ref. [6] evaluated the performance of their method using a dataset of driver video sequences and found that the proposed method could accurately detect drowsiness with a high degree of precision. The findings of [1] suggested that this method had the potential to be used in real-world applications to improve road safety and reduce the risk of drowsy driving accidents. Moon et al. conducted research on this topic using a different approach. They proposed a CNN-based method to automatically detect and analyze eye-blink frequency counts in real-time video sequences of drivers. The main contribution of this research was the use of CNNs for the detection of eye-blink-based driver drowsiness. The proposed method was trained using a large dataset of eye-blink features from both drowsy and non-drowsy drivers. The authors found that the proposed CNN-based method could accurately detect drowsiness with a high degree of precision compared with traditional drowsiness detection methods. The performance of the proposed method was evaluated under various scenarios. These included changes in lighting conditions and driver behavior. Jain et al. [8] investigated a method to detect driver drowsiness based on an analysis of eye-blink features and used support vector machine (SVM) classification to determine the level of drowsiness. An SVM can be an effective approach for eye-blink detection because it can handle non-linear boundary problems and is robust against overfitting. However, to maintain the superior performance of the SVM classifier in eye-blink detection, it is important to perform high-quality feature extractions from eye images and obtain details of the size of the training dataset and the choice of SVM hyperparameters.

Various high-accuracy methods have been proposed for drowsiness detection such as electrooculography (EOG) and infrared camera-based techniques. In our proposed method, we not only focused on the eye aspect ratio (EAR) method to detect blinks but also applied facial landmark points for driver yawning [11]. We evaluated an OpenCV-based blink-detection system and simultaneously applied media-pipe facial landmarks for a single input eye image and yawning state measurement to alert drowsy drivers. This study makes the following contributions to the field of driver drowsiness detection.

1. Our proposed novel method detected driver drowsiness or fatigue in real time using eye-blinking and yawning classes. Our approach applied computer-vision and deep learning techniques to detect and track eye-blinks and yawns in real time.
2. We introduced a custom dataset consisting of images and videos of drivers in various drowsy and alert states by combining eye-blink measurements and yawning class coordinates. This dataset included the annotation coordinates of eye-blinking and yawning classes, which enabled researchers to train and test drowsiness detection algorithms.
3. We evaluated our method using both simulated and real-time world data, demonstrating its high accuracy in detecting driver drowsiness.
4. We provide a detailed analysis of the features most indicative of drowsy behavior, including the duration and frequency of eye-blinks and yawns as well as the variability in these features over time.

**Figure 1:**
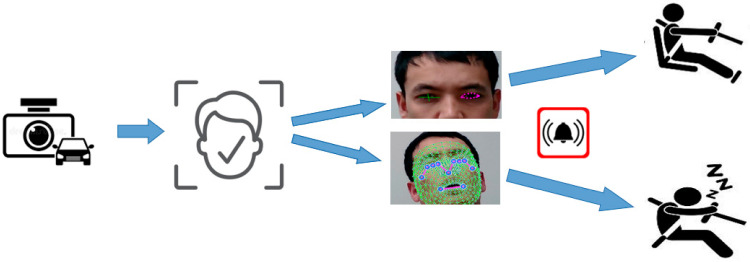
Workflow of proposed method.

Overall, our research represents a significant advance in the field of drowsiness detection, and the proposed approach has important practical applications in improving road safety and preventing accidents caused by fatigued driving, as shown in Figure 1.

The figure depicts the framework of our proposed drowsiness detection approach, which included collecting data, extracting relevant features, preprocessing data, selecting the most important features (especially from eye-tracking-based and yawning-based landmarks), training a machine-learning model, and evaluating the model performance by integrating it with other systems.

The remainder of this paper is organized as follows. Section 1 provides an overall introduction to the field of study and the proposed approach. Section 2 includes techniques and relevant publications on drowsiness detection. Section 3 describes our proposed method from data acquisition to the testing and evaluation process. Section 4 analyzes the experimental results by comparing them with other algorithms. Section 5 concludes the study and discusses the limitations of our research.

## 2. Related Work

Eye-movement detection refers to the process of detecting and measuring eye movements. Eye movements provide important information regarding the attention, perception, and cognitive processes of participants by analyzing saccades, fixations, and smooth pursuit. Eye-movement detection can be applied to the study of visual and auditory processing, learning and memory, and other behavioral aspects of human performance. Eye-movement detection can be achieved using several techniques, including EOG, infrared (IR) eye trackers, video-based eye trackers, and dual-purpose tracking.

### 2.1. EOG

This is a non-invasive method that measures the electrical potential difference between the cornea and retina to detect eye movements. This difference arises because the cornea is positively charged with respect to the retina and the overall potential difference changes when the eye moves. The electrical signal generated by eye movements can be used to detect the direction and magnitude of eye movements and can also be applied to study the underlying physiological processes with respect to eye-movement control. EOG is widely applied in psychology, neuroscience, and ophthalmology for visual perception attention and cognitive processes. Moreover, EOG can be applied to human–computer interactions, where eye movements control the computer cursor or interact with graphical interfaces.

A few studies [12,13,14] have focused on the EOG technique, in which the authors focused on eye-movement-related research. In [12], an overview of the history, basic principles, and applications of EOG is provided, together with some limitations of the EOG technique for eye-movement measurements. In [13], the development of a new EOG recording system and its application to the study of eye movements are described. In [14], a new low-noise amplifier for EOG signals is presented and its performance is compared with those of other amplifier designs. They evaluate the performance of the amplifier in terms of its noise, bandwidth, and distortion and then compare it with other amplifier designs that are commonly utilized for EOG signals. The experimental results indicated that the proposed amplifier design provided better performance than existing designs in terms of a lower noise, a wider bandwidth, and lower distortion rates. The origin of the EOG as a technique to measure eye movements can be traced back to several pioneering studies. In 1950, Harold Hoffman first developed the EOG system and used it to study eye movements and visual perception. Steiner extended Hoffman’s work and developed a more sophisticated EOG system capable of measuring either horizontal or vertical movements. In the 1960s, Bowen et al. developed a mathematical model of EOG that enabled a more accurate and detailed description of the electrical signals generated by eye movements. Currently, EOG is widely used in several fields and plays a crucial role in advancing our knowledge of visual perception, attention, and cognitive processes.

### 2.2. Infrared (IR) Eye Tracking

IR eye-tracking techniques involve the use of infrared light to illuminate the eyes and a camera to capture the reflection of light in the eyes. IR works by using an infrared light source in the eyes and capturing the reflection using a camera. IR eye tracking is widely used in fields such as psychology, HCI, market research, and gaming. This is because IR eye-tracking techniques are non-intrusive and can be used with a wide range of subjects such as those wearing glasses and contact lenses [15].

### 2.3. Video-Based Eye Tracking

In computer-vision-based eye tracking and eye detection from an input image, localization is considered to be the main area. These two areas are challenging because there are several issues associated with eye detection and tracking such as the degree of eye openness or the variability of eye sizes in target objects. Computer-vision-based methods use video cameras to capture images of the eyes and analyze them to determine eye movements. Cameras can be set up to track eye movements in real-time or in laboratory settings. The basic idea of this method is to use image processing techniques to detect and track the position of the pupils in a video stream and then use the acquired information to infer the direction of gaze. Generally, there are two types of video-based eye-tracking systems, remote and wearable. Regarding the remote-tracking type, eye tracking uses a camera placed at a certain distance from the participant and then records their eye movements at the same time. In the wearable eye-tracking type, a camera is attached to a headset or glasses that the participant wears and then records the participant’s eye movements from a relatively greater proximity. The benefit of this technique is that a more complete picture of the gaze behavior is obtained. In recent years, several researchers [14,15,16,17,18] have discussed video-based eye-tracking movements by analyzing eye recordings. In [16], a method for real-time video-based eye tracking using deep learning algorithms that combined CNNs and RNNs to accurately track eye positions in real time was proposed. The authors evaluated their system using a publicly available dataset. The experimental results showed that the proposed method outperformed traditional approaches in terms of accuracy, speed, and robustness. Eye-tracking pattern recognition has achieved remarkable results. The authors of [19] proposed a principal component analysis (PCA) model to identify six principal components. The identified components were used to reduce the dimensionality of the image pixels. The authors used an ANN to classify pupil positions where calibration was required to observe five varied points that all represented different pupil positions. Lui et al. [20] presented both eye-detection and -tracking methods. Researchers have applied the Viola–Jones face detector Haar features to locate the face in an input image. The template matching (TM) method was used for eye detection. TM is widely used in computer vision because it can be applied to object recognition, image registration, and motion analysis. For eye-detection purposes, a similarity measure was computed based on metrics such as cross-correlation, mean-squared error, and normalized correlation. The authors of [20] used different methods for eye detection and tracking such as Zernike moments (ZMs) to extract the rotation invariant of eye characteristics and an SVM for eye or non-eye classification.

### 2.4. Dual-Purpose Tracking

This method combines infrared and video-based tracking techniques to improve the accuracy of eye-movement detection. Infrared tracking provides highly accurate eye positions, whereas video-based tracking provides additional information regarding eye appearance and motion. Huang et al. [21] presented an algorithm to detect eye pupils based on eye intensity, size, and shape. In this study, the intensity of the eye pupil was applied as the main feature in pupil detection and the SVM identified the location of the eye. In pupil-fitting, corneal reflection and energy-controlled iterative curve-fitting methods are efficient approaches, as reported by Li and Wee [22]. For pupil boundary detection, an ellipse-fitting algorithm can be used, which is controlled by the energy function. In the ellipse-fitting process, the task is to find the best fit for a given data point and to minimize the distance between the input data points and the sum of the squared distances.

### 2.5. Yawning-Based Drowsiness Detection

Yawning is a physical reflex lasting 4–7 s of gradual mouth gaping and a rapid expiratory phase with muscle relaxation [23]. As it is a natural physiological response to tiredness, it is widely used in research to identify drowsy drivers. The authors of [24] proposed a method based on tracking the condition of the driver’s mouth and recognizing the yawning state. They implemented a cascade-boosted classifier for Haar wavelet features on several different scales if those positions were measured by Canny integral images. The AdaBoost algorithm was used for feature selection and localization. To determine the yawning condition of the driver, an SVM was applied to the model prediction in the case of the data instances for model testing. For this, the SVM was trained using mouth- and yawning-related images by transforming the data and scaling was performed on the data by applying the radial function kernel. In [25], the researchers proposed a yawning detection approach that included measurements of eye-closure duration. They calculated the eye state and yawning coordinate measurements based on mouth conditions. For mouth detection, [25] used a spatial fuzzy c-means (s-FCM) clustering method.

The authors also applied an SVM for drowsiness detection; the input of the SVM was the width-to-height ratios of the eyes and mouth. Calculations were performed based on the state of the eye and mouth of the driver such as whether the eye was half-open or closed or yawning or not. The final classification results were used to determine whether the driver was in a dangerous condition. Ying et al. [26] determined driver fatigue or drowsiness by monitoring variances in eye and mouth positions. This method relied on skin-color extraction. To find the state of the moving object, a back propagation (BP) neural network was required that enabled the recognition of the object’s position [27]. A similar approach by Wang et al. [28] mentioned that the mouth region was located in terms of multi-threshold binarization in intensity space using the Gaussian model in the range of RGB color space. By applying the lip corners to the integral projection of the mouth in the vertical direction, the lower and upper lip boundaries were highlighted as the openness of the mouth. Therefore, the yawning stage was determined by the degree of mouth opening, with respect to the ratio of the mouth–bounding rectangle. When the ratio in the box identified a large open mouth over a predefined threshold for a continuous number of frames, the driver was classified as drowsy.

## 3. Proposed Method

**Figure 2:**
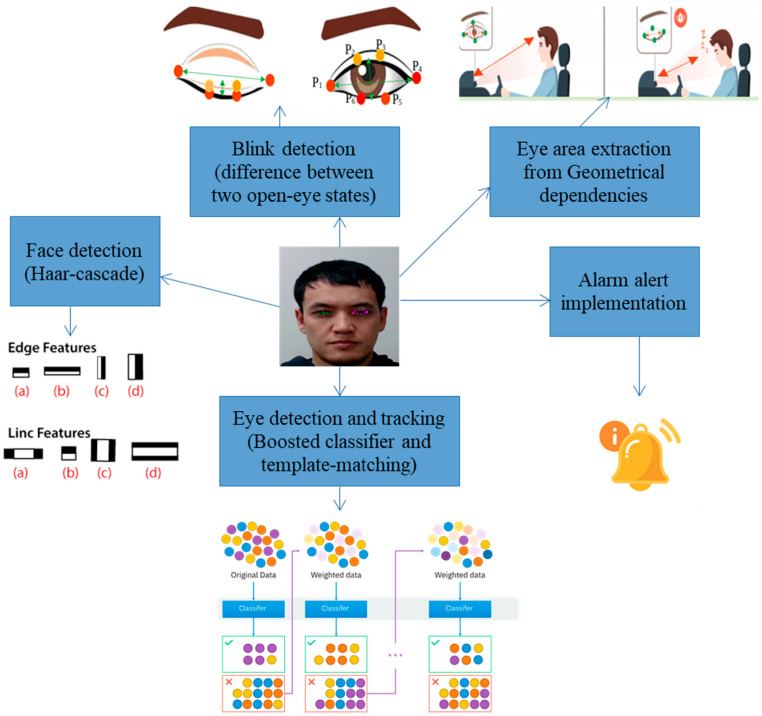
Workflow of the proposed method with algorithm application, eye tracking, and distance measurement of eye states and between the camera and driver’s head location.

Our study proposed the detection of driver drowsiness by simultaneously calculating eye-blinks and yawning. The drowsiness of the driver was classified as “drowsy” in terms of three categories. First, yawning-based drowsiness levels were identified. Next, eye-blinking-based drowsiness was identified. Finally, a class was obtained as a combination of yawning- and eye-blinking-based classes, as shown in Figure 2.

Threshold-based blink detection was applied to determine whether the eyes were open or closed. A drowsiness evaluation was performed in three phases: (1) eye-blink detection, based on landmark coordinates; (2) metric computation; and (3) a drowsiness index estimation. In addition, we added iris-tracking DL algorithms to increase the accuracy of the blink estimation model. Iris detection provides information on open eyes. When the eye is closed, the algorithm classifies it as a “closed eye”. Therefore, if “closed eye” and landmark metric computation match in classification, then it is highly predicted that the model has more “true-positives”, which indicates better model performance. In other words, without an iris-detection-based blink measurement, the model may show less accuracy. For example, there will be incorrect blink counts due to misclassifications or false estimations in the illustration of looking down and the actual closure of the eyes. Consequently, these false-positives may lead to an increase in the overestimated drowsy alerts. Using the medium-pipe face mesh model, we detected the face and eyes of the driver. As the requirement for drowsiness detection focused only on the driver, the detection algorithms were set to detect only a single face. Based on this, the boundary landmarks of the left and right eyes were detected. The drowsiness detection model obtained measurements based on the distances of the eye landmark movements. In other words, the driver mostly made upward and backward movements while driving. Therefore, leaning changes in the driver should not have affected the distance-based calculation of blink detection. Similarly, the value was kept at the same amount and we considered drivers to be blinking based on that value. To improve the blink-detection performance of the model, we set the average distance between the camera location and the driver from 0.3 m to 0.6 m. To normalize the distance changes of the driver, we considered the ratio of eyelids with regard to the eye size [29].

### Midpoint Calculation

**Figure 3:**
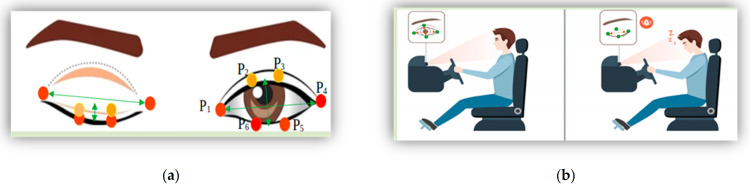
(**a**) Landmark detection eye-state measurement and (**b**) face detection, eye tracking, and analyses.

Regarding anatomical landmarks, the midpoint of the eye is situated approximately half-way between the medial and lateral corners of the eye where the upper and lower eyelids meet each other. However, it is important to mention that the state of the midpoint of the eye can vary among individuals and with gaze direction. According to [21], the midpoint is the middle or center of a line segment. Once the required points were identified, we set the blink calculation by algorithms between the upper and lower eyelid meeting points, as shown in Figure 3.

The eye-blink-detection method is a computer-vision and human–computer interaction approach used to measure the frequency of eye-blinking and to detect variations in blink patterns. One of the most commonly and widely used methods for eye-blink calculations is eye detection and tracking. In this method, computer-vision techniques and deep learning algorithms track the eyes in real time. Based on the detected eye, the blink frequency is calculated based on the disappearance and reappearance of the eyelids, as shown in Table 1 and Table 2.

Electrooculography (EOG) is another technique widely used to measure eye movements by recording the electrical potential differences produced by eye movements. The EOG technique records the electrical activity of eye muscles, in which a signal is generated by the movement of the eyes and EOG is measured using electrodes placed near the outer corner of the eye and near the temple. The EOG method utilizes small electrodes placed near the eyes to detect the electrical signals produced by retinal movement. The retina is the innermost layer of the eye and contains photoreceptor cells such as rods and cones. Retinal movement does not occur on its own; rather, the eye muscles move the entire eye to change the direction of gaze and the position of the retina relative to the visual scene. EOG is commonly used in the fields of neuroscience, human–computer interaction, and cognitive psychology for eye-movement studies, tracking eye-blink frequencies, and measuring eye-gaze patterns [22]. Overall, this method is inexpensive, can be used to study eye movements in real time, and provides a high temporal and spatial resolution.

## 4. Experimental Results and Discussions

### 4.1. Dataset

**Figure 4:**
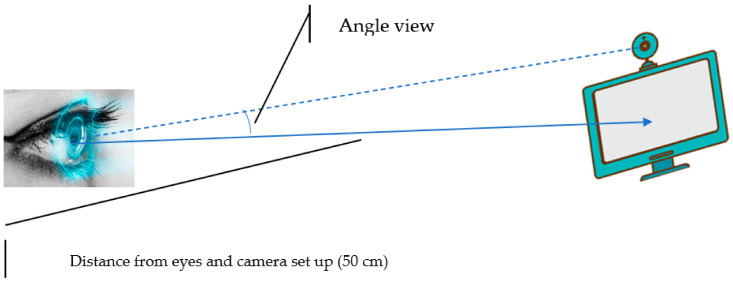
Environmental illustration for drowsiness detection.

**Figure 5:**
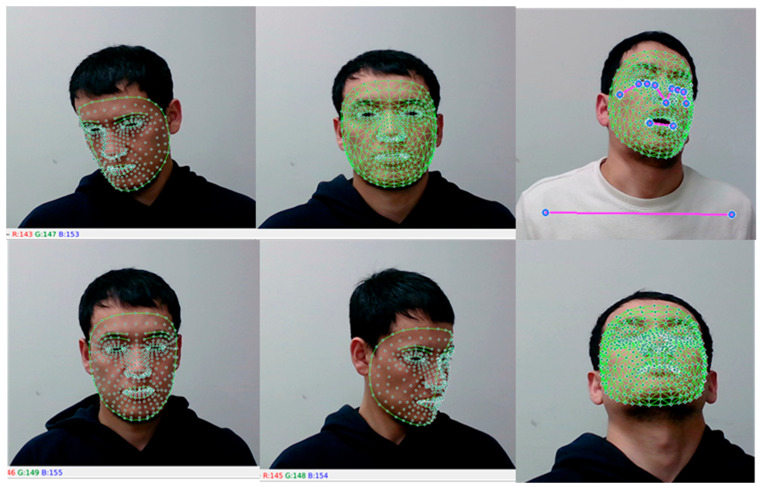
Data collection process.

In this section, we present the experimental set up of the proposed method, as shown in Figure 4 and Figure 5.

Based on the purpose of this study, our goal was to collect landmark-related datasets to analyze driver drowsiness. For that, we collected six class datasets by considering the position of the driver’s drowsy state. The six classes included the driver falling right, falling left, and falling back as well as open-eye and closed-eye states. To train the model with only one eye, we first gained weights for the blink-detection model. We trained the Haar–Cascade classifier to detect faces. After detection, we captured the coordinates of facial landmarks and exported them to a comma-separated value (csv) file, based on the proposed classes. After the detection model was trained, we applied landmarks of the eyes and specified classes to that model. Landmarks were adjusted to relative classes. The model was trained on multi-class classification model in order to understand the relationship between the driver states and representative coordinates [30].

### 4.2. Media-Pipe Model

Facial landmarks were crucial to the proposed approach. Therefore, we used a media-pipe framework, which is a machine-learning pipeline designed to process time-series data in real time. The medium-pipe provides approximately 16 open-source prebuilt TensorFlow and TF-Lite models. Generally, a medium-pipe consists of three main parts: a framework for inference from sensory data; a set of tools for the performance evaluation; and a collection of reusable inference and processing components [31]. In our case, the face mesh model was best-suited to extracting driver eye landmarks in real time. The face mesh was a machine-learning model developed by transfer learning on top of a blaze face model.

### 4.3. Evaluation Metrics

There are a few examples of performance metrics and the choice of the metric depends on the specific issue, nature of the data, and purpose of the analysis. The metrics help to determine how well the proposed approach or model performs. The general acceptance of agreement with true facts can be calculated in terms of the computation of correctly recognized class numbers (true-positive; TP), the number of correctly recognized examples that do not depend on the class (true-negative; TN), and examples that were either incorrectly assigned a class (false-positive; FP) or that were not recognized as class examples (false-negative; FN), as in our previously published papers [32,33,34,35,36].

(1) $$\mathrm{Precision}=\frac{\mathrm{TP}}{\mathrm{TP}+\mathrm{FP}}$$

(2) $$\mathrm{Recall}=\frac{\mathrm{TP}}{\mathrm{TP}+\mathrm{FN}}$$

(3) $$F\text{-}1\;\mathrm{score}=\frac{2*Recall*Precision}{Recall+Precision}$$

**Figure 6:**
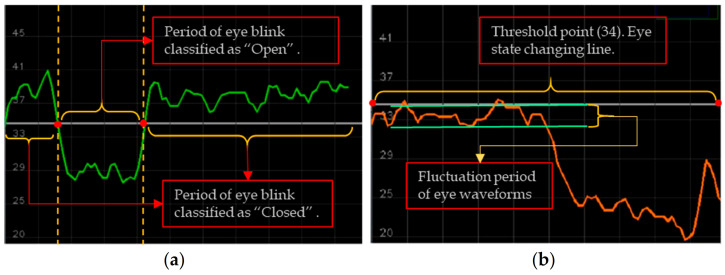
Eye waveform representations: (**a**) blinking fluctuation between classes; (**b**) classifier line and stable “Open” eye class example.

To analyze eye movements in open and closed forms, we set a blink differentiation threshold by measuring the midpoint based on the EAR scalar value. The EAR scalar value represented the calculation of eye opening and closing, as shown in Figure 6a. The scalar value could be calculated by substituting six landmark coordinates located around the eye, as shown in Equation (4).

(4) $$\mathrm{EAR}=\frac{∥P_{2}-P_{6}∥+∥P_{3}-P_{5}∥}{2∥P_{1}-P_{4}∥}$$

Equation (4) describes the EAR measurement, where P_n_ represents the landmark positions on the retina. P_2_, P_3_, P_5_, and P_6_ points were applied to measure the height of the eye, while P_1_ and P_4_ points were used to identify the width of the eye. The equation showed that, over time, the EAR value fluctuated upward and downward; accordingly, blinking waveforms of the eyes were produced. The proposed approach detected both eye landmarks and performed calculations using Equation (4). The eye-state decision was made based on the distance calculation between landmarks. “Open” and “Closed” eye states were denoted by two different colors; pink for “Open” and green for “Closed”. The upper and lower threshold points were set as 45 and 20, respectively. To determine an appropriate threshold value for the waveforms of eye coefficients, we applied the standard deviation of the coefficients. The standard deviation measured the dispersion or variability of a set of values related to eye-blinks. Applying Equation (4), the waveforms of the eye fluctuation line were set to 34 where the eye state was determined or changed from one class to another, as shown in Figure 6.

The local modulus maximum points were related to blinks where the local maximum points were set as positive if M(k) = 1 and negative if M(k) = −1. Every local module’s maximum and minimum points represented only a single point to determine the upward and downward waveforms and set a threshold. The sequence line was generated using eye landmark-annotated movement data (x_n_ and y_n_). The initial value set to the threshold was S(k) = 1; otherwise, S(k) = 0. The threshold T_S_ was estimated using the standard deviation of the first-level wavelet coefficients, as in the equation below:

(5) $$S(k)=\left\{\begin{array}{c} 1,\left|\omega \left(k\right)\geq T_{S}\right|\\ 0,\left|\omega \left(k\right)<T_{S}\right| \end{array}\right.$$

From the incorporated threshold-depicted continuous wave line points of landmark-based eye-movement blinking analysis, the open-eye or closed-eye classes indicated not-drowsy or drowsy states, respectively. Expression S(k) illustrated a function resulting in the output of either 1 or 0, based on the value of (ω ) by comparing it with a threshold value of T_S_. If the value of $\omega \left(k\right)$ was greater than or equal to the threshold value of T_S_, then S(k) = 1 and the function outputted 1 when the values of the threshold and conditions were met. Similarly, if the value of $\omega \left(k\right)$ was less than the threshold value of T_S_, then S(k) = 0 and the function outputted 0 because of the lower value $\omega \left(k\right)$ and the condition matched.

Table 3 represents the configuration of the software and hardware of this research.

**Figure 7:**
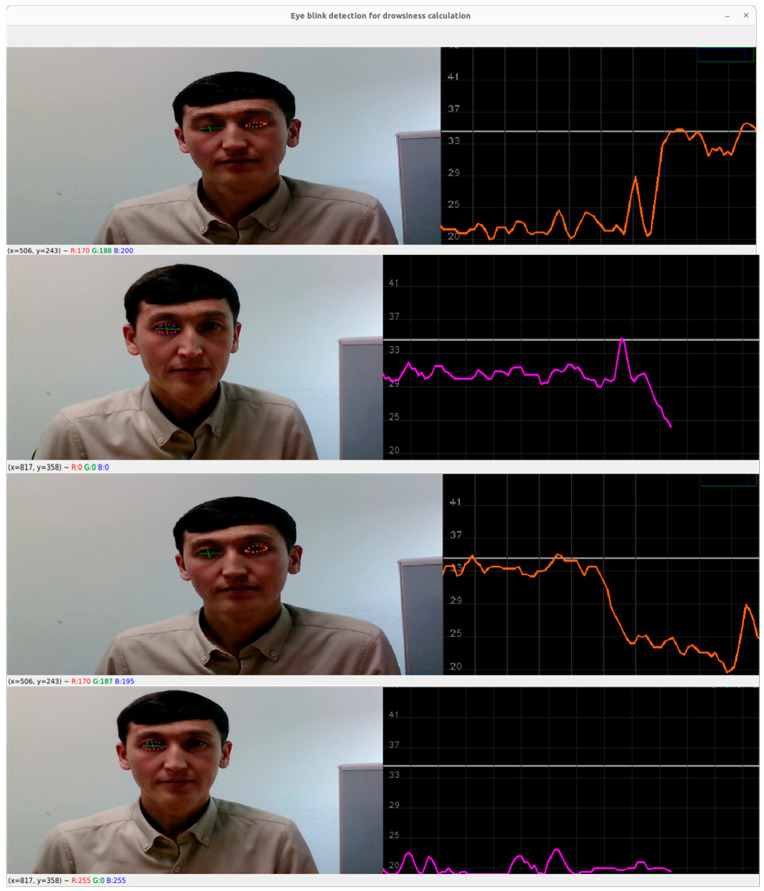
Eye tracking and EAR measurements.

Figure 7 depicts the eye-tracking-based driver eye-state analysis according to Equation (1). The threshold line was set to point 34, where the upper waveforms were classified as “Closed” eyes and the lower ones represented “Open” eyes. From the pictures, we observed that when the eyes were closed, the wave lines increased; when the eyes were close to being closed or were open, the wave line fluctuated around the threshold. Additionally, when the eyes were wide-open, the eye wave rate decreased to point 20.

**Figure 8:**
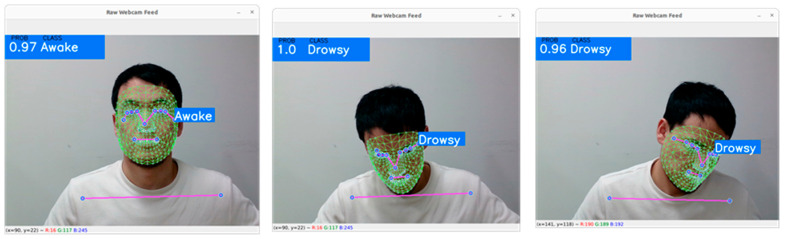

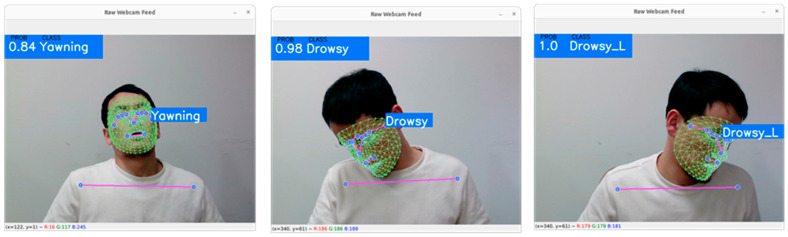
Driver state measurement based on landmark analysis.

Figure 8 shows the experimental results of landmark-based driver drowsiness detection. The accuracy of driver drowsiness detection was superior in the head-leaning cases (above 96%). The yawning class, in which the driver’s eyes were closed and the mouth was open, achieved 85% accuracy.

## 5. Limitations

The main idea of the study was to detect drowsy drivers by analyzing their face and tracking their eyes. The proposed approach may not be relevant for drivers who wear sunglasses. Having sunglasses may interrupt the model’s ability to detect eye landmarks; it cannot then measure eye-blinks. In other words, eye-blink analyses were performed by face detection; the landmark implementation to the face was specifically applied to both eyes of the driver. As drivers wear sunglasses and other objects may be present that interrupt the facial landmarks, the model may become confused when tracking and analyzing the state of the driver. In further research, we will focus on the development of the proposed method as well as searching to find better ways to solve eye-tracking problems when drivers wear sunglasses [37,38,39].

## 6. Conclusions

In this study, we proposed a method to detect drowsiness by combining multiple algorithms. The first step involved the collection of facial landmark coordinates from custom data. We gathered data for different classes, including awake-open eyes, drowsy-closed eyes (with the head leaning to the right, left, and front), and yawning. The driver’s drowsiness was solely determined by calculating landmark coordinates. The authors did not specify the exact details of the calculations, but it appeared that they used facial landmarks to analyze the driver’s state. To further enhance drowsiness detection, we proposed a real-time eye-tracking approach. We used eye-gaze landmarks to measure blinking. Blinking was quantified using the EAR equation and a threshold was set to classify eye wave line fluctuations into two classes: “Open” (indicating that the driver was not drowsy) and “Closed” (indicating drowsiness). By combining both algorithms, the facial landmark-based approach and eye-tracking approach, the proposed method improved the estimation of the driver’s drowsiness state. We believe that a combination of these algorithms will lead to more accurate and reliable drowsiness detection. However, additional details regarding specific algorithms and their performance metrics are required to fully evaluate the effectiveness of the proposed approach.

The figures show that when the eyes were closed, the wave line increased. When the eyes were close to being closed or open, the wave line fluctuated around the threshold line, showing a drowsy driver and not a drowsy driver, respectively. When the eyes were wide-open, the eye-wave rate decreased to point 20. This study reported the accuracy of drowsiness detection in different scenarios. The detection accuracy for cases in which the driver was leaning their head was above 96%. For the yawning class, where the driver’s eyes were closed and the mouth was open, the overall accuracy reached 85%. Without further details and evaluation metrics such as the sample size, validation methodology, and statistical analysis, it is challenging to fully assess the reliability and generalizability of the reported accuracy values.

## Figures and Tables

**Table 1:** Proposed method class measurements.

Yawning-Based Drowsiness Detection	Eye-Blinking-Based Drowsiness Detection		Joint Yawning and Eye-Blinking Drowsiness Detection
Landmark-based measurement	Landmark coordinate-based eye-closure measurement	Iris detection-based eye-closure measurement	Combination of landmark coordinates and eye-gaze landmarks with blinking measurements

**Table 2:** Comparison of the proposed method with similar studies.

Driver Drowsiness Detection Studies	Characteristics of Approaches		
	Sensing Method	Algorithm Class	Embedded Algorithms
Ellicie-Healthy [15]	IR-sensor-based	Threshold-based	MCU
You et al. [10]	Camera-based	DL-based	No
Sharan et al. [18]	Camera-based	DL-based	Raspberry Pi
Kim et al. [19]	Camera-based	DL-based	No
Our method	Camera-based	Threshold + DL-based	

**Table 3:** Software and hardware configuration.

Software	Programming Language	Python
	OS	Linux
**Hardware**	CPU	AMD Ryzen Threadripper 1900X 8-Core Processor 3.80 GHz
	GPU	Titan Xp 32 GB
	RAM	128

## Data Availability

Not applicable.
